# Intermittent Theta-Burst Stimulation Increases the Working Memory Capacity of Methamphetamine Addicts

**DOI:** 10.3390/brainsci12091212

**Published:** 2022-09-08

**Authors:** Yurong Sun, Huimin Wang, Yixuan Ku

**Affiliations:** 1 School of Psychology and Cognitive Science, East China Normal University, Shanghai 200062, China; 2 Center for Brain and Mental Well-Being, Department of Psychology, Sun Yat-sen University, Guangzhou 510275, China; 3 Peng Cheng Laboratory, Shenzhen 518066, China

**Keywords:** theta-burst stimulation, methamphetamine use disorder, working memory

## Abstract

The present study aimed to explore the effect of intermittent theta-burst stimulation (iTBS) on visual working memory for people suffering from methamphetamine use disorder (MUD). Five sessions of iTBS were carried over the left dorsolateral prefrontal cortex (DLPFC) or the vertex as a sham control, with each session in one day. Orientation free-recall tasks were conducted before the iTBS stimulation, after the first and fifth sessions of stimulation. Results showed that when compared with the sham group, a single session of iTBS over the left DLPFC improved participants’ working memory performance. Specifically, iTBS over the left DLPFC increased the working memory capacity and such effects enlarged with multiple sessions. The present finding suggested that iTBS over DLPFC could be a promising intervention method to enhance the cognitive function of addicts with MUD.

## 1. Introduction

Drug abuse is a serious, relapsing mental disorder which causes considerable monetary and societal problems. Among the abused drugs, Methamphetamine (MA) is the most prevalent one worldwide. Recently, animal and human studies have revealed the neural mechanisms underlying methamphetamine use disorder (MUD). Chronic MA abuse on the one hand leads to structural and functional deficits of the reward-related dopamine system that influences activity in the orbital frontal cortex (OFC), which then results in an extremely higher reward expectation for MA-related stimuli and reduced interest to other stimuli [1,2]. On the other hand, impairment of cognitive function in the dorsolateral prefrontal cortex (DLPFC) is a non-negligible consequence of MUD [3,4]. Taken together, the frontal disorder caused by MA abuse results in abnormal reward expectation, reduced impulse control and impaired executive function to inhibit addictive behaviors [5].

Working memory (WM), serving as the short-term storage and manipulation of information, is one crucial component of executive control, which relies on the prefrontal function [6], and the functioning of the dopamine reward system. Indeed, dopamine release determines WM functioning [7], and in reverse, WM training leads to more dopamine release [8]. At the same time, WM deficits have been found in dopamine-deficient groups such as addicts [9,10,11]. Specifically, at the behavioral level, addicts with poorer WM performance showed stronger cue-evoked cravings than those with higher WM performance [12]. At the neural level, it has been suggested that the stronger the activation in WM-relevant brain regions (the frontal–parietal network) under the WM task is, the lower the relapse rate after withdrawal from alcohol will be [13]. Furthermore, WM training has been shown to alleviate alcohol and drug abuse behaviors [14,15]. Thus, intervention targeting WM and prefrontal functions might be useful for alleviating addictive behaviors.

Recent studies have emphasized the role of repetitive transcranial magnetic stimulation (rTMS) as a safe and convenient way to treat mental disorders [16,17,18]. Traditionally, high frequency (>5 Hz) rTMS leads to excitatory effects and low frequency (≤1 Hz) rTMS leads to inhibitory effects [19]. To shorten the stimulating time, Theta Burst Stimulation (TBS) has been developed, including both excitatory intermittent TBS (iTBS) and inhibitory continuous TBS (cTBS; [20,21]).

Excitatory rTMS targeting on the DLPFC has been suggested as a promising intervention method to decrease the drug intake, craving, and relapse rates in addicts [22,23,24,25]. There are two possible mechanisms underlying TMS benefit. Firstly, excitatory rTMS on the prefrontal cortex directly affects the dopamine system by promoting/boosting the release of dopamine in the striatum [26], and regulating dopamine in the bilateral anterior cingulate cortex (ACC) and OFC [27]. Secondly, excitatory stimulation over the DLPFC increases the ability of WM, which tightly correlates with impulsive control and executive function; enhanced impulsive control and executive function then lead to a better inhibition of craving and impulsive behaviors. Assessing WM performance in addicts could reveal the possible mechanism. Interestingly, a new study using spatial n-back tasks to assess WM has found that excitatory iTBS over DLPFC indeed promotes WM performance in MA addicts [28]. However, this study used multiple sessions (20 in total) of stimulation over the DLPFC and has not yet revealed how many sessions of stimulation could produce this enhancement, limiting future applications.

Therefore, in the current study, we assessed the excitatory iTBS effects on visual WM in addicts with MUD. We used a free-recall task, which is more sensitive in measuring WM than n-back and change-detection tasks [29]. Multiple sessions of iTBS on the DLPFC were carried out over MUD. To explore whether a single session of stimulation would be enough to produce changes in WM, or whether multiple sessions would be required to obtain a significant effect, the current study contained five intervention sessions, once per day for five days, and measured WM performance after the first and the fifth stimulations respectively. We hypothesized that iTBS targeting the DLPFC would improve visuospatial WM performance continuously.

## 2. Materials and Methods

### 2.1. Participants

Eighteen male addicts (mean age 32.35 ± 3.06 years) with a regular use of MA (weekly or daily use for 2–15 years) took part in the experiment. All of them were recruited from Nanchang drug rehabilitation center and met the diagnostic criteria of substance use disorder in the DSM-5. Participants were neurologically healthy, right-handed individuals with normal or corrected-to-normal vision. They participated in the study voluntarily and signed the written informed consent approved by the Institutional Review Board of East China Normal University and Shanghai Mental Health Center before the experiment.

Participants were randomly assigned to two groups: iTBS and sham groups. One participant was excluded from the following analysis because his performance in the WM task was at the chance level (guess rate > 50%). Thus, there were 9 participants in the iTBS group and 8 in the sham group. After conducting an independent samples *t*-test, no differences were detected in age, education years, addiction years, current abstinence duration, monthly dosage, and baseline WM performance between the two groups (Table 1).

### 2.2. Behavioral Measurements

**Figure 1:**
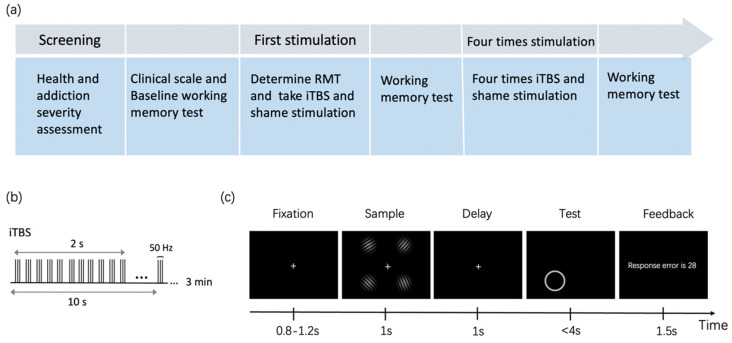
(**a**) Flowchart of the whole experiment; (**b**) iTBS protocol; (**c**) The procedure of the free-recall working memory task.

A free-recall WM task was used to measure the cognitive function of addicts. As shown in Figure 1c, the screen firstly showed a fixation for 0.8–1.2 s, then displayed four Gabor patches for 1 s. The Gabor Patches were randomly generated and the orientations of every two of them were differentiated by at least 10°. Participants were asked to remember the orientations of every Gabor patch as accurately as possible. After that, a blank screen was presented as the retention interval for 1 s. At the test phase, a circular ring indicating the probe item would be shown in one of the four quadrants. Participants were asked to recall the orientation of the probed Gabor Patch by moving the mouse to the circle and clicking on a certain location that would represent the orientation of the previously shown Gabor patch.

Stimuli were generated and presented using Psychtoolbox (Matlab Psychtoolbox-3; psychtoolbox.org). All of the stimuli were displayed on the black background of an LCD laptop with a resolution of 1024 × 768. The refresh rate of the screen was 60 Hz. To facilitate the detection of orientations, we used elongated high-contrast (75%) and fixed phase Gabor patches (gratings with spatial frequency of 4.5 c/deg and standard deviations of 0.17 and 0.34). Gabor patches were shown at four fixed positions in each quadrant, distanced by 2.1 eccentricities from the fixation dot.

The task procedure is shown in Figure 1a. The baseline WM task contained 60 trials and lasted for 6 min. The second WM task containing 300 trials was conducted after the first session of brain stimulation. The third WM task containing 300 trials was done after five sessions of brain stimulation. 

### 2.3. TMS Procedures

TMS treatments were delivered by the Yiruide CCY-IA TMS machine, with a figure-of-eight coil targeting the left DLPFC and vertex. The stimulation site was defined by a hat based on the international 10–20 system, in which F3 represented the left DLPFC. Before the theta-burst stimulation, we first determined the Resting Motor Threshold (RMT) of the left motor cortex with the minimum strength needed to elicit right-hand finger movement in 5 out of 10 trials. The amplitude of iTBS was applied with 80% RMT. All participants received five sessions of stimulation in one week, each session for one day. As illustrated in Figure 1b, one session of intervention consisted of 600 pulses: triplets of stimulation on 50 Hz, repeating every 200 ms, with 2 s on and 8 s off for 3 min. For the sham group, the same stimulation parameters were adopted, but the coil was placed at a perpendicular angle to the vertex.

### 2.4. Data Analysis and Statistics

Statistical analysis was conducted with SPSS and R. To compare the group differences in demographic and clinical characteristics, independent sample t-tests were conducted. Then, the mean response error and mean reaction time (RT) were calculated for each group separately. The response error was the absolute angular difference between the presented orientation and the reported orientation (ranging from 0 to 90 degrees). We performed a Linear Mixed Model (LMM) analysis to investigate the intervention effect on response error and RT respectively. In this model, the group (with two levels: iTBS and sham) and stimulation (with three levels: pre stimulation, after one session of stimulation and after five sessions of stimulation) were treated as fixed factors, and the subjects were included as the random factor. 

We also performed the classic analysis of covariance (ANCOVA) to investigate the intervention effect after one session iTBS and after five sessions iTBS, respectively. The response error or RT before the stimulation (pre-test) was treated as a covariant and the group (iTBS and shame) was included as the fixed factor. 

**Figure 2:**
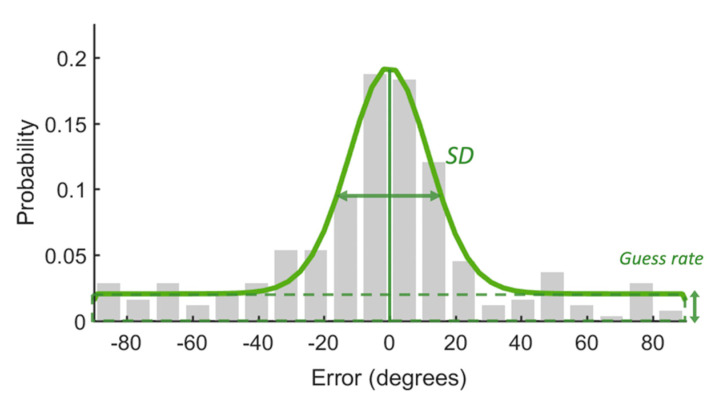
An example of response error distribution. Most response errors are centered around zero. The standard deviation of the distribution indicates the precision of WM. The guess rate represents the proportion of responses that were guessed randomly. The guess rate could be used to calculate capacity.

In addition, we fitted the response error data with the Standard Mixture Model (SMM; [30]) to further estimate the capacity of WM and the quality of remembered items. The model assumed that some items within the capacity limit in the memory array were remembered with a certain precision, but the others were not remembered at all. The latter would be guessed randomly for recall. As shown in Figure 2, the response error would be fitted with a circular Gaussian-shaped model with two parts: a von Mises distribution that described the remembered items and a uniform distribution that captured the guess part of the response error. Parameter g was the height of the uniform distribution which could be used to calculate the memory capacity (K). K was obtained by multiplying the probability at which the probed item was remembered (1 g) with the set size. The standard deviation (SD) of the von Mises distribution is regarded as memory precision. 

## 3. Results

As shown in Table 1, no differences in age and education years were found between the iTBS and sham groups. Meanwhile, there were no significant differences between the two groups in clinical characteristics including addiction years, abstinent days, dosage per month, and sleep quality. 

**Figure 3:**
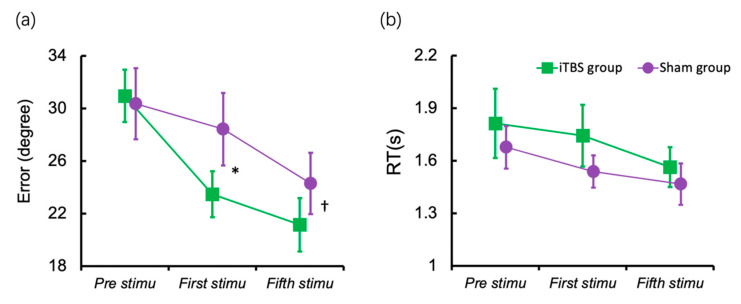
Working memory performance before and after stimulation. (**a**) Mean response error of the baseline session (before stimulation), after the first stimulation and after the fifth stimulation; (**b**) Mean RT at the baseline session, after the first stimulation and after the fifth stimulation. Error bar indicates Standard Error (SE) of the mean. † *p* < 0.1, * *p* < 0.05.

The intervention’s effects on the response error after the first session of stimulation were examined using Linear Mixed Model (LMM). Figure 3a displayed the results for the response errors. There was a significant main effect of the stimulation session (F (2, 30) = 37.377, *p* < 0.001) and a non-significant main effect of the group (F (1, 15) = 0.677, *p* = 0.424). However, the interaction between the sessions of stimulation and group was significant (F (2, 30) = 4.765, *p* = 0.016), which meant that the differences between groups changed over sessions. Then, we compared the group difference after the first session and the fifth session separately using ANCOVA, with the baseline performance as the covariate. The iTBS group indicated a lower response error than the sham group after the first session (F (1, 14) = 8.398, *p* = 0.012, η_p_² = 0.375). The group difference in response error after the fifth session was marginally significant (F (1, 14) = 3.750, *p* = 0.073, η_p_² = 0.211). 

Figure 3b displayed the results for RT; the main effect of session of stimulation (F (2, 30) = 3.739, *p* = 0.035) was significant. However, the main effect of the group (F (1, 15) = 0.694, *p* = 0.418) and the interaction between sessions and group (F (2, 30) = 0.211, *p* = 0.881) were not significant. 

**Figure 4:**
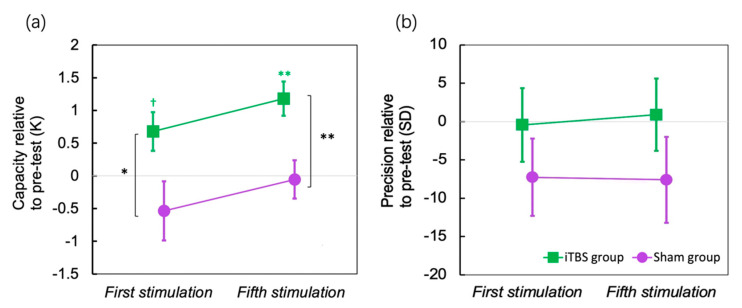
Standard Mixture Model results. (**a**) capacity change relative to baseline after one session’s stimulation and after five sessions of stimulation; (**b**) memory precision change relative to baseline after one session’s stimulation and after five sessions’ stimulation. The colored star symbols mean significant difference from zero. Error bar indicates SE of the mean. † *p* < 0.1, * *p* < 0.05, ** *p* < 0.01.

The results of the standard mixture model are shown in Figure 4 (Figure 4a for capacity and Figure 4b for precision). To compare the change by iTBS, the performance before stimulation was used as a baseline to be subtracted. The capacity was increased for about 0.676 items after the first session of iTBS (*p* = 0.051, Cohen’s d = 0.766), and for about 1.176 items after the fifth session of iTBS (*p* = 0.002, Cohen’s d = 1.503), yet the precision did not change over sessions. In contrast, the sham stimulation did not change either the capacity or precision.

## 4. Discussion

The present study used excitatory iTBS over the left DLPFC on methamphetamine addicts, while measuring their WM performance before the stimulation, after the first session of stimulation, and after five sessions of stimulation. When compared with sham stimulation on the vertex, a significant improvement in the overall WM performance was found following only one session of iTBS on DLPFC. However, such an improvement was not enlarged following five sessions of iTBS on DLPFC. In addition, we fitted response errors with the SMM [30] to calculate the capacity of WM and the precision of remembered items separately. A single session of iTBS over DLPFC increased the capacity by about 0.676 items and five sessions of iTBS continuously increased the capacity by about 1.176 items.

The prefrontal cortex has been supposed to be highly engaged in WM processing. The first neural evidence of WM-related cells was identified in the DLPFC [31] and WM functions depend on activity in the DLPFC [32,33]. Critically, we found that although there was a learning effect in the behavior of the overall WM performance for the sham group, the stimulating group had a significantly larger enhancement for the behavior. After only one single session of stimulation, the two groups had significant differences in WM performance, while in the previous research, enhancement was observed after 20 sessions of stimulation [28], but whether one session worked remained unknown. Our present findings thus greatly increase the feasibility of using iTBS to modulate the cognitive function of MA addicts. However, a similar paradigm as our designs would be useful to detect subtle changes in WM performance.

Using a change-detection task, Wang et al. [34] have found that excitatory anodal direct current stimulation (tDCS) on the DLPFC increases the visual WM capacity of normal participants measured with a change-detection task. In MA addicts, and by applying a continuous free-recall task that more precisely measures WM performance, the present study consistently found that excitatory iTBS over the DLPFC increases WM performance as well as the capacity measures. Interestingly, multiple sessions of iTBS increased the capacity continuously, and after five sessions of iTBS over the DLPFC, the addicts’ mean capacity for WM could be increased for more than one item, which was substantial, since WM capacity for normal participants was three to four [35]. WM capacity is fundamental as it has been proven to be correlated with general intelligence [36] and to be involved in successful self-regulation, executive behavior and impulse control [37]. Thus, such an increase in WM capacity might lead to better cognitive control capability in addicts that would inhibit unwanted cravings and relapses. Indeed, a plethora of research has investigated the effect of stimulating the DLPFC in treating addiction and some of them have had promising effects [23]. From the results of the current study, the possible mechanism of high-frequency rTMS and iTBS in treating addiction could be through increased WM capacity and promoted prefrontal functions. However, it should be noted that the stimulation could also promote dopamine projection to the DLPFC from the reward system [38,39]. Either way, stimulation of the DLPFC would be a promising intervention method for drug addictions. 

We did not observe changes in WM precision of remembered items after the stimulation. Our previous studies have proven that visual WM precision and high fidelity representations are maintained in visual areas [40,41]. The posterior parietal cortex (PPC) has also been suggested to represent precise images [42,43]. Future studies are needed to apply stimulation on the visual cortices or PPC to examine the potential changes in precision in addicts.

The current study was carried out shortly before COVID-19 and the number of participants was not so large, since entering the center became difficult. Although some statistical results were marginal, most of the effect sizes were substantial, and we still found robust and consistent results when compared with previous research. Yet, we did not validly measure subjects’ craving for MA, and thus were unable to correlate increased or decreased WM performance with the changes in craving score. In this case, it is hard to investigate the relationship between WM performance and addiction severity. Future studies could adopt multiple craving measurements to obtain a more accurate craving score, and explore the relationship between changes in craving score and changes in WM precision before and after stimulation. 

## 5. Conclusions

In conclusion, the current study indicated that both a single session and five sessions of iTBS on left DLPFC significantly improved WM performance in MA addicts. Specifically, the stimulation increased visual WM capacity. 

## Figures and Tables

**Table 1:** Demographic characteristics of participants.

Subject Variable	iTBS Group (*N* = 9)	Sham Group (*N* = 8)	*t*	*p*
	*M* (S.D.)	*M* (S.D.)		
Age	32.333 (3.536)	32.375 (2.669)	0.027	0.979
Education years	8.667 (4.213)	9.563 (2.382)	0.530	0.604
Abstinent days	272.444 (97.815)	373.625 (249.258)	1.128	0.277
Addiction years	7.111 (4.106)	7.875 (3.482)	0.411	0.687
Dosage per month (g)	13.844 (12.838)	6.725 (4.742)	−1.739	0.103
PSQI	9.111 (2.369)	7.25 (3.412)	−1.319	0.207
BIS	97 (16.606)	104.75 (22.601)	0.812	0.429
Baseline response error (degree)	30.960 (5.989)	30.314 (7.613)	−0.195	0.848
Baseline RT (s)	1.813 (0.592)	1.672 (0.341)	−0.590	0.564

PSQI: Pittsburgh Sleep Quality Index; BIS: Barratt Impulsiveness Scale; *t*: *t* Value of independent samples *t*-test; *p*: *p* Value.

## Data Availability

Data sharing not applicable.
